# Development and Validation of a New Prognostic System for Patients with Hepatocellular Carcinoma

**DOI:** 10.1371/journal.pmed.1002006

**Published:** 2016-04-26

**Authors:** Fabio Farinati, Alessandro Vitale, Gaya Spolverato, Timothy M. Pawlik, Teh-la Huo, Yun-Hsuan Lee, Anna Chiara Frigo, Anna Giacomin, Edoardo G. Giannini, Francesca Ciccarese, Fabio Piscaglia, Gian Lodovico Rapaccini, Mariella Di Marco, Eugenio Caturelli, Marco Zoli, Franco Borzio, Giuseppe Cabibbo, Martina Felder, Rodolfo Sacco, Filomena Morisco, Elisabetta Biasini, Francesco Giuseppe Foschi, Antonio Gasbarrini, Gianluca Svegliati Baroni, Roberto Virdone, Alberto Masotto, Franco Trevisani, Umberto Cillo, ITA.LI.CA study group

**Affiliations:** 1 Department of Surgery, Oncology and Gastroenterology, University of Padua, Padua, Italy; 2 Department of Surgery, Johns Hopkins Hospital, Baltimore, Maryland, United States of America; 3 National Yang-Ming University, Taipei City, Taiwan; 4 Department of Medicine, Taipei Veterans General Hospital, Taipei City, Taiwan; 5 Biostatistics Unit, University of Padua, Padua, Italy; 6 Gastroenterology Unit, Department of Internal Medicine, IRCCS Azienda Ospedaliera Universitaria San Martino IST, University of Genoa, Genoa, Italy; 7 Division of Surgery, San Marco Hospital, Zingonia, Italy; 8 Division of Internal Medicine, Department of Medical and Surgical Sciences, Alma Mater Studiorum, University of Bologna, Bologna, Italy; 9 Division of Internal Medicine and Gastroenterology, Complesso Integrato Columbus, Università Cattolica del Sacro Cuore, Rome, Italy; 10 Division of Medicine, Bolognini Hospital, Seriate, Italy; 11 Division of Gastroenterology, Belcolle Hospital, Viterbo, Italy; 12 Division of Radiology, Department of Medicine, Fatebenefratelli Hospital, Milan, Italy; 13 Division of Gastroenterology, Biomedical Department of Internal and Specialistic Medicine, University of Palermo, Palermo, Italy; 14 Division of Gastroenterology, Bolzano Regional Hospital, Bolzano, Italy; 15 Division of Gastroenterology and Metabolic Diseases, University Hospital of Pisa, Pisa, Italy; 16 Division of Gastroenterology, Department of Medicine and Surgery, University of Naples Federico II, Naples, Italy; 17 Division of Infectious Diseases and Hepatology, Azienda Ospedaliero–Universitaria di Parma, Parma, Italy; 18 Department of Internal Medicine, Ospedale per gli Infermi di Faenza, Faenza, Italy; 19 Division of Internal Medicine and Gastroenterology, Fondazione Policlinico Universitario A. Gemelli, Università Cattolica del Sacro Cuore, Roma, Italy; 20 Division of Gastroenterology, Polytechnic University of Marche, Ancona, Italy; 21 Division of Internal Medicine 2, Ospedali Riuniti Villa Sofia Cervello, Palermo, Italy; 22 Gastroenterology Unit, Ospedale Sacro Cuore Don Calabria, Negrar, Italy; 23 Division of Semeiotics, Department of Medical and Surgical Sciences, Alma Mater Studiorum, University of Bologna, Bologna, Italy; University of Texas Southwestern Medical Center, UNITED STATES

## Abstract

**Background:**

Prognostic assessment in patients with hepatocellular carcinoma (HCC) remains controversial. Using the Italian Liver Cancer (ITA.LI.CA) database as a training set, we sought to develop and validate a new prognostic system for patients with HCC.

**Methods and Findings:**

Prospective collected databases from Italy (training cohort, *n* = 3,628; internal validation cohort, *n* = 1,555) and Taiwan (external validation cohort, *n* = 2,651) were used to develop the ITA.LI.CA prognostic system. We first defined ITA.LI.CA stages (0, A, B1, B2, B3, C) using only tumor characteristics (largest tumor diameter, number of nodules, intra- and extrahepatic macroscopic vascular invasion, extrahepatic metastases). A parametric multivariable survival model was then used to calculate the relative prognostic value of ITA.LI.CA tumor stage, Eastern Cooperative Oncology Group (ECOG) performance status, Child–Pugh score (CPS), and alpha-fetoprotein (AFP) in predicting individual survival. Based on the model results, an ITA.LI.CA integrated prognostic score (from 0 to 13 points) was constructed, and its prognostic power compared with that of other integrated systems (BCLC, HKLC, MESIAH, CLIP, JIS). Median follow-up was 58 mo for Italian patients (interquartile range, 26–106 mo) and 39 mo for Taiwanese patients (interquartile range, 12–61 mo).

The ITA.LI.CA integrated prognostic score showed optimal discrimination and calibration abilities in Italian patients. Observed median survival in the training and internal validation sets was 57 and 61 mo, respectively, in quartile 1 (ITA.LI.CA score ≤ 1), 43 and 38 mo in quartile 2 (ITA.LI.CA score 2–3), 23 and 23 mo in quartile 3 (ITA.LI.CA score 4–5), and 9 and 8 mo in quartile 4 (ITA.LI.CA score > 5). Observed and predicted median survival in the training and internal validation sets largely coincided. Although observed and predicted survival estimations were significantly lower (log-rank test, *p* < 0.001) in Italian than in Taiwanese patients, the ITA.LI.CA score maintained very high discrimination and calibration features also in the external validation cohort.

The concordance index (C index) of the ITA.LI.CA score in the internal and external validation cohorts was 0.71 and 0.78, respectively. The ITA.LI.CA score’s prognostic ability was significantly better (*p <* 0.001) than that of BCLC stage (respective C indexes of 0.64 and 0.73), CLIP score (0.68 and 0.75), JIS stage (0.67 and 0.70), MESIAH score (0.69 and 0.77), and HKLC stage (0.68 and 0.75). The main limitations of this study are its retrospective nature and the intrinsically significant differences between the Taiwanese and Italian groups.

**Conclusions:**

The ITA.LI.CA prognostic system includes both a tumor staging—stratifying patients with HCC into six main stages (0, A, B1, B2, B3, and C)—and a prognostic score—integrating ITA.LI.CA tumor staging, CPS, ECOG performance status, and AFP. The ITA.LI.CA prognostic system shows a strong ability to predict individual survival in European and Asian populations.

## Abstract 2

Using Italian and Taiwanese cohorts, Alessandro Vitale and colleagues develop and validate a staging system and prognostic model for hepatocellular carcinoma.

## Editors' Summary

**Background:**

Primary liver cancer—a tumor that starts when a liver cell acquires genetic changes that allow it and its descendants to divide uncontrollably and move around the body (metastasize)—is the sixth most common cancer and the second leading cause of cancer-related deaths worldwide. Liver cancer kills more than three-quarters of a million people every year, mostly in resource-limited countries. The risk of developing hepatocellular carcinoma (HCC; the most common type of liver cancer) is highest in eastern and southeastern Asia; among wealthier nations, the risk of HCC is particularly high in Italy. HCC can be treated by surgical removal of part of the liver, liver transplantation, ablation (which uses an electric current to destroy the cancer cells), intra-arterial therapies (which deliver drugs directly into the liver), or systemic (whole body) drug therapies. However, the symptoms of HCC, which include weight loss, tiredness, and jaundice, are vague. HCC is therefore rarely diagnosed before the cancer is advanced and has a poor prognosis (likely outcome)—fewer than 5% of patients survive for five or more years after diagnosis.

**Why Was This Study Done?:**

Cancer staging describes the severity of a cancer based on the size and extent of the original tumor and whether the tumor has metastasized. Staging helps doctors estimate the patient’s prognosis and can help them devise a treatment plan that will, hopefully, improve patients’ quality of life and may extend their life expectancy. Several staging systems have been devised for HCC, but prognostic assessment of patients with HCC is controversial. No single prognostic model (a model that allows clinicians to obtain predictions about the likely outcomes of individual patients) has been universally adopted. An ideal model is difficult to achieve as it would need to consider tumor-related, liver-function-related, and patient-related variables, all of which have different impacts on patient prognosis. Here, the researchers use a database created by the Italian Liver Cancer (ITA.LI.CA) group that includes information on more than 5,000 Italians with HCC to develop a new prognostic model to predict individual patient outcomes based on tumor-related, liver-function-related, and patient-related variables.

**What Did the Researchers Do and Find?:**

The researchers first defined ITA.LI.CA stages for HCC using tumor characteristics only. They then used information on 3,628 patients in the ITA.LI.CA database (the “training” set) and statistical modeling to calculate the relative prognostic value of tumor staging, Eastern Cooperative Oncology Group (ECOG) performance status (an indicator of whether patients are able to look after themselves and undertake normal daily activities), liver function (measured using the Child—Pugh score), and alpha-fetoprotein level (a liver tumor marker) in the prediction of the survival of individual patients. Based on these modeling results, they constructed an ITA.LI.CA integrated prognostic score. The researchers report that the observed and predicted median (average) survival times in the training set and in an internal validation cohort of 1,555 additional patients in the ITA.LI.CA database were similar. Moreover, although the observed and predicted survival times were lower in the Italian patients than in 2,651 patients with HCC from Taiwan, the ITA.LI.CA score had high discrimination and calibration features in this external validation cohort as well (the discrimination of a prognostic model indicates its ability to separate patients into groups with different outcomes, the calibration of a prognostic model is the degree of correspondence between predicted and observed outcomes). Finally, the prognostic ability of the new ITA.LI.CA prognostic model was significantly better than that of several other prognostic scoring systems.

**What Do These Findings Mean?:**

These findings introduce a revised staging system for HCC and an integrated prognostic score—the ITA.LI.CA prognostic score—based on this staging system, Child—Pugh score, ECOG performance status, and alpha-fetoprotein level that has a greater ability to predict survival among Italian and Taiwanese patients than previous prognostic models. Because this study was retrospective—previously recorded data, including outcomes, were used to develop the prognostic model—a prospective trial is needed to validate the ITA.LI.CA prognostic score. That is, researchers need to enroll a group of patients, determine their ITA.LI.CA prognostic scores, and then follow the patients to determine their actual outcomes. If validated in this way and in other populations, use of the ITA.LI.CA prognostic score should allow clinicians to provide more accurate prognoses for individual patients, and may be a starting point for evaluating which treatment option is best suited to each patient presenting with HCC.

**Additional Information:**

This list of resources contains links that can be accessed when viewing the PDF on a device or via the online version of the article at http://dx.doi.org/10.1371/journal.pmed.1002006.

- This study is further discussed in a *PLOS Medicine* Perspective by Neehar Parikh and Amit Singal
- The US National Cancer Institute provides information about all aspects of cancer, including detailed information for patients and professionals about primary liver cancer and about cancer staging (in English and Spanish)
- The American Cancer Society also provides information about liver cancer (including information on support programs and services; available in several languages)
- The UK National Health Service Choices website provides information about primary liver cancer (including a video about coping with cancer) and about cancer staging
- Cancer Research UK (a not-for-profit organization) provides detailed information about primary liver cancer
- The British Liver Trust (a not-for-profit organization) also provides information about liver cancer, including a personal story
- MedlinePlus provides links to further resources about liver cancer (in English and Spanish)

## Introduction

Liver cancer is the sixth most common cancer and the second most common cause of cancer death worldwide, leading to nearly 746,000 deaths in 2012 [1]. Hepatocellular carcinoma (HCC) is largely a problem in low- and middle-income countries and regions, where 83% (50% in China alone) of the estimated 782,000 new HCC cases occurred in 2012 [1]. In addition, in recent decades, HCC incidence and HCC-related deaths have been rising, not only in low- and middle-income countries but also in high-income countries [2]. The highest HCC risk, measured by age-standardized rates and classified as “very high risk,” has been recorded in eastern (31.9) and in southeastern Asia (22.2). The HCC risk in Italy (11.0) was classified as “moderately high risk,” and it was one of the highest in high-income countries [1].

Over the last 20 years, a variety of prognostic systems have been proposed in an attempt to address the interrelationship of prognostic factors among HCC patients and the complexity of HCC treatment [3–17]. In the literature, several HCC prognostic systems have been described but no single system for HCC has been universally adopted. Some systems—such as Barcelona Clinic Liver Cancer (BCLC) staging, United Network for Organ Sharing (UNOS) tumor node metastases (TNM) staging, Hong Kong Liver Cancer (HKLC) staging, and American Joint Committee on Cancer (AJCC) TNM staging—are “staging classification” schemas, typically based on systematic reviews of the literature and/or expert opinions [6–9]. These systems stratify the HCC population in stages exclusively or mainly defined by tumor characteristics. The aim of these schemas is usually to link stages to guidelines for the management of patients with HCC and the design of clinical trials. However, these systems often lack prognostic power [10] or strong statistical support such as external validation [8].

Other systems—such as the Model to Estimate Survival in Ambulatory HCC Patients (MESIAH), Cancer of the Liver Italian Program (CLIP), and Japanese Integrated Staging (JIS) scores—are “conventional” prognostic scores that incorporate variables that were significant in multivariable Cox survival analyses [11–17]. The strength of these scores consists in objective and reproducible variables and rigorous statistical methodology. Unfortunately, even if these prognostic scores correlate well with outcome, often they are not suitably generalizable to populations different from the one that generated the score, and they don’t define tumor stages easily usable in clinical practice [11–17].

Based on these considerations, we sought to develop and validate a new prognostic system for HCC patients including both a tumor staging and a prognostic score. The Italian Liver Cancer (ITA.LI.CA) dataset, including more than 5,000 Italian HCC patients prospectively enrolled from 1 January 1987 to 31 March 2012, was used for the training and internal validation sets. A large HCC population from Taiwan served as the external validation cohort, to test the prognostic value of this new system in an Asian population.

## Methods

### Study Groups

Prospectively collected data of 5,290 consecutive patients with HCC, each managed in one of the 19 institutions participating in the ITA.LI.CA study group and enrolled between 1 January 1987 and 31 March 2012 were analyzed.

The institutional review boards of the participating institutions approved the study. According to the Italian and Taiwanese laws, no patient approval is needed for retrospective studies. Informed consent was obtained as usual for medical, surgical, and radiological treatments, but not specifically for patient data to be used in this retrospective study. Patients gave written consent for every procedure performed in the hospitals, including use of data for medical purposes.

Details about patient data collected for this study are described in S1 Text. For each patient the following composite variables were calculated and recorded: Child—Pugh score (CPS), BCLC stage, UNOS modified TNM stage, HKLC stage, CLIP score, JIS stage, MESIAH score, and modified BCLC stage [6–8,12,15,17–20]. After exclusion of 107 cases without complete follow-up data or lost to follow-up, a total of 5,183 patients were included in the analysis and randomly allocated into the training or test (internal validation) set in an approximately 2:1 ratio (3,628:1,555). In addition, in order to test the general application of our scheme to HCC patients in another center, an external validation was performed in a cohort of 2,651 patients from Taipei (Taiwan) with HCC diagnosed between 1 January 2002 and 31 December 2012. In the Taiwanese cohort, there were no patients lost to follow-up or with incomplete follow-up data.

### Descriptive Statistics

Baseline characteristics were examined based on frequency distribution; continuous data are presented as median (interquartile range) unless indicated otherwise. Univariate comparisons were assessed using Student’s *t* test, Wilcoxon rank-sum test, or chi-squared test as appropriate. Missing data relative to study covariates always involved less than 10% of patients. Thus, missing values were imputed using the maximum likelihood estimation method [21].

Overall survival was defined from the date of first diagnosis of HCC to the date of death, last follow-up evaluation, or data censoring (31 December 2012). Kaplan—Meier survival curves were used to estimate median overall survival and the 1-, 3-, and 5-y overall survival rates. The survival curves were also stratified according to ITA.LI.CA prognostic system quartiles, and the log-rank test was used to compare differences in survival.

Specific statistical analyses [22] were conducted to develop and validate the ITA.LI.CA prognostic system. Here we describe only the rationale behind this process and the main statistical analyses, while more statistical details are described in S2 and S3 Texts.

### Development of the ITA.LI.CA Prognostic System

We first defined the ITA.LI.CA tumor staging as a composite variable based on four main stages: 0 (very early), A (early), B (intermediate), and C (advanced) (Table 1). These stages were similar to the BCLC stages but were designed to have some important differences from the BCLC system so as to avoid several confusing aspects of that system. First, ITA.LI.CA stages are based only on tumor characteristics (i.e., Eastern Cooperative Oncology Group (ECOG) performance status test (PST) and CPS did not contribute to stage definition). Second, single tumor >5 cm was considered B stage, regardless of the treatment received. Third, based on published data and clinical knowledge [7,8,23], B stage patients were further stratified into three sub-stages: B1, single HCC >5 cm or 2–3 nodules measuring 3–5 cm; B2, 2–3 nodules measuring >5 cm or >3 nodules measuring ≤5 cm; B3, >3 nodules measuring >5 cm or presence of intrahepatic vascular invasion. Stage C included only HCC patients with extrahepatic vascular invasion or metastases [23].

**Table 1:** The ITA.LI.CA tumor staging system.

Diameter of the Largest Nodule (cm)	Number of Nodules	Vascular Invasion or Metastases	Stage
≤2	1	No	0
≤3	2–3	No	A
2–5	1	No	A
3–5	2–3	No	B1
>5	1	No	B1
>5	2–3	No	B2
≤5	>3	No	B2
>5	>3	No	B3
Any	Any	Intrahepatic	B3
Any	Any	Extrahepatic	C

BCLC staging [6] and UNOS modified TNM staging [7] have been taken as reference: stage 0, very early; stage A, early; stage B, intermediate; stage C, advanced. Based on recent evidence [6,16], a sub-classification of stage B was introduced.

Similar to in BCLC and HKLC staging [6,8], established variables that have determinative roles in prognostic assessment of HCC patients—ITA.LI.CA tumor staging, CPS, ECOG PST, and alpha-fetoprotein (AFP)—were selected a priori in building the prognostic system. AFP was included in the ITA.LI.CA system because of its known prognostic relevance [12] and new evidence suggesting its utility as an exclusion criterion for liver transplantation [24,25]. AFP was added as a categorical variable (AFP > or ≤ 1,000 μg/l); 1,000 μg/l was used as the cutoff because this value represented the AFP threshold yielding the best prognostic discrimination in the multivariate survival model (better also than AFP used as a continuous variable).

To develop the ITA.LI.CA integrated system for individual prognostic prediction, we selected overall survival as the outcome of interest. We therefore modeled overall survival based on the above four prognostic factors (i.e., ITA.LI.CA tumor staging, CPS, ECOG PST, and AFP) using a multivariable survival model to account for their relative effects.

### Exploratory Analyses

As explained in S2 Text and S1 and S2 Figs, a multivariable parametric model was chosen to construct the prognostic score in the training set.

### Performance Assessment of the ITA.LI.CA Prognostic System

The performance of the ITA.LI.CA prognostic system was assessed based on a rigorous validation methodology (through internal validation and external validation in a large population from Taiwan). To compare the prognostic performance of the ITA.LI.CA prognostic score with that of other systems, we calculated the Akaike information criterion (AIC), the concordance index (C index), and the test for trend chi-square [22,26]. The lower the AIC value, the higher the discriminatory ability of the staging system. The higher the C index and the test for trend chi-square, the higher the discriminatory ability and monotonicity of gradients of the staging system. To measure whether the performance of the ITA.LI.CA score was significantly better than that of other systems we used the likelihood ratio test.

Due to the long enrollment period (from 1987 to 2012) of the ITA.LI.CA population, a subgroup analysis for time period was also performed, to evaluate and overcome potential time-related biases.

## Results

### Characteristics of the Study Groups

The characteristics of the study groups are presented in Table 2. There were no differences in the baseline characteristics of the training set and internal validation set. The median age of the three groups (training, internal validation, and external validation) was 68, 67, and 65 y, respectively; in all groups, there was a preponderance of male gender (75%, 76%, and 78%, respectively). As expected, the majority of patients in the Taiwan validation group were hepatitis B carriers (55%), whereas in the Italian cohort, the majority of patients were hepatitis C carriers (61%). Liver function was better preserved in the external validation group (median Model for End-Stage Liver Disease [MELD] score = 8) than in the Italian cohort (median MELD score = 11). While the number of tumors was similar among the three groups, patients from Taiwan were more likely to have larger tumors and vascular invasion. Taiwanese patients also tended to have a high ECOG PST, compared with the two Italian sets. As expected, hepatic resection was the most common therapeutic approach in the Taiwanese cohort [20].

**Table 2:** Patient characteristics of the three groups.

Variable	Training Set (*n* = 3,628)	Internal Validation Set (*n* = 1,555)	External Validation Set (*n* = 2,651)
**Sex** *			
Male	2,724 (75%)	1,189 (76%)	2,054 (78%)
Female	904 (25%)	366 (24%)	597 (22%)
Age (years)	68 (60–74)	67 (61–74)	65 (55–75)
**HBV+** *	632 (17%)	253 (16%)	1,462 (55%)
**HCV+** *	2,199 (61%)	961 (62%)	812 (31%)
**Alcohol abuse** *	959 (26%)	417 (27%)	485 (18%)
**Albumin (g/l)**	36 (32–39)	35 (32–39)	37 (32–41)
**Bilirubin (μmol/l)** *	22.2 (15.4–32.5)	22.2 (15.4–32.5)	15.4 (10.3–23.9)
**International normalized ratio** *	1.3 (1.1–1.5)	1.3 (1.1–1.5)	1.1 (1–1.1)
**Sodium (mmol/l)**	139 (137–140)	139 (137–140)	139 (136–141)
**Creatinine (μmol/l)**	79.6 (70.7–97.2)	79.6 (70.7–97.2)	88.4 (70.7–106.1)
**Presence of ascites** *	963 (27%)	425 (27%)	645 (24%)
**Presence of encephalopathy** *	222 (6%)	90 (5%)	83 (3%)
**AFP (μg/l)** *	23 (8–100)	25 (8–113)	49 (9–860)
**CPS** *	6 (5–7)	6 (5–7)	5 (5–7)
**MELD score** *	11 (9–14)	11 (9–14)	8 (7–11)
**Diameter of the largest lesion (mm)** *	30 (20–43)	30 (20–46)	45 (25–90)
**Multinodular (>3 nodules)**	794 (22%)	347 (22%)	593 (22%)
**Macroscopic vascular invasion**			
Any*	478 (13%)	215 (14%)	928 (35%)
Intrahepatic	211 (6%)	107 (7%)	n.d.
Extrahepatic	267 (7%)	108 (7%)	n.d.
**Presence of metastases**	95 (3%)	41 (3%)	0 (0%)
**BCLC stage**			
0	261 (7%)	108 (7%)	162 (6%)
A*	1,181 (33%)	487 (31%)	585 (22%)
B	448 (12%)	228 (15%)	348 (13%)
C	1,511 (42%)	632 (41%)	1,170 (44%)
D*	227 (6%)	100 (6%)	385 (15%)
**Modified BCLC stage**			
0	261 (7%)	108 (7%)	162 (6%)
A*	1,181 (33%)	487 (31%)	585 (22%)
B	1,106 (30%)	499 (32%)	823 (31%)
C	853 (24%)	361 (24%)	696 (26%)
D*	227 (6%)	100 (6%)	385 (15%)
**ECOG PST**			
0*	2,182 (60%)	955 (61%)	1,491 (56%)
1	851 (24%)	353 (23%)	496 (19%)
2	483 (13%)	195 (13%)	335 (13%)
3–4*	112 (3%)	52 (3%)	329 (12%)
**Main treatment**			
Resection*	392 (11%)	162 (10%)	704 (27%)
Transplantation	71 (2%)	35 (2%)	0 (0%)
Ablation*	1,073 (30%)	446 (29%)	511 (19%)
Intra-arterial therapy	944 (26%)	427 (27%)	784 (29%)
Sorafenib	112 (3%)	48 (3%)	0 (0%)
Other systemic treatment	307 (8%)	130 (8%)	77 (3%)
Best supportive care	729 (20%)	307 (20%)	575 (22%)

Data are presented as number (percent) or median (interquartile range).

*Statistically significant difference between the Italian (training and internal validation) and Taiwanese (external validation) study groups.

HCV+, hepatitis C virus positive; HBV+, hepatitis B virus positive; n.d., not determined.

### The ITA.LI.CA Prognostic System

Median duration of follow-up was 58 mo for the Italian patients (interquartile range, 26–106 mo) and 39 mo for the Taiwanese patients (interquartile range, 12–61 mo). Overall survival was significantly lower (log-rank test, *p <* 0.001) in the Italian patients than in the Taiwanese patients (S3 Fig). For the Italian patients, the median overall survival was 32 mo (interquartile range, 14–64 mo), and the 1-, 3-, and 5-y overall survival rates were 80%, 48%, and 29%, respectively. For Taiwanese patients, the median overall survival was 57 mo (interquartile range, 15–104 mo), and the 1-, 3-, and 5-y overall survival rates were 78%, 61%, and 49%, respectively.

In building the model, four main prognostic factors were selected: ITA.LI.CA tumor staging, ECOG PST, CPS, and AFP (Table 3). These variables had the following impact in determining the final score. For ITA.LI.CA tumor staging, one point was assigned for each increase in stage (from 0 to 5 for 0, A, B1, B2, B3, and C). For CPS to define liver function, zero points were assigned for a CPS of 5, one point for a CPS of 6 or 7, two points for a CPS of 8 or 9, and three points for a CPS > 10. For ECOG PST, zero points were assigned for a PST of 0, one point for a PST of 1–2, and three points for a PST of 3–4. CPS and PST points together formed the ITA.LI.CA functional score. For AFP level, two points were assigned for AFP > 1,000 μg/l.

**Table 3:** Development of the ITA.LI.CA prognostic system.

Prognostic Factor	Stage, Score or Value	Estimate* ± Standard Error	*p*-Value	Points**
**ITA.LI.CA tumor staging**	0	0		0
	A	0.36 ± 0.08	<0.001	1
	B1	0.48 ± 0.14	<0.001	2
	B2	0.92 ± 0.21	<0.001	3
	B3	1.11 ± 0.29	<0.001	4
	C	1.40 ± 0.39	<0.001	5
**ITA.LI.CA functional score**				
CPS score	5	0		0
	6	0.17 ± 0.06	0.0029	1
	7	0.31 ± 0.13	<0.001	1
	8	0.52 ± 0.20	<0.001	2
	9	0.56 ± 0.29	<0.001	2
	10–15	0.75 ± 0.39	<0.001	3
ECOG PST	0	0		0
	1	0.21 ± 0.05	<0.001	1
	2	0.41 ± 0.12	<0.001	1
	3–4	0.86 ± 0.24	<0.001	3
**AFP (μg/l)**	≤1,000	0		0
	>1,000	0.59 ± 0.07	<0.001	2

*Multivariable survival parametric model estimate.

**Points = estimate × 3.5, rounded.

The lowest score (ITA.LI.CA score = 0) of the model corresponded to the best prognosis, and the highest score (ITA.LI.CA score = 13) was associated with the worst prognosis (S2 Table; *p <* 0.001 at log-rank test). To test the prognostic calibration of the ITA.LI.CA score, patients were divided into quartiles at the 25th, 50th, and 75th percentiles of the risk score. Quartile 1 coincided with ITA.LI.CA score ≤ 1, quartile 2 with score 2–3, quartile 3 with score 4–5, and quartile 4 with score > 5.

**Fig 1:**
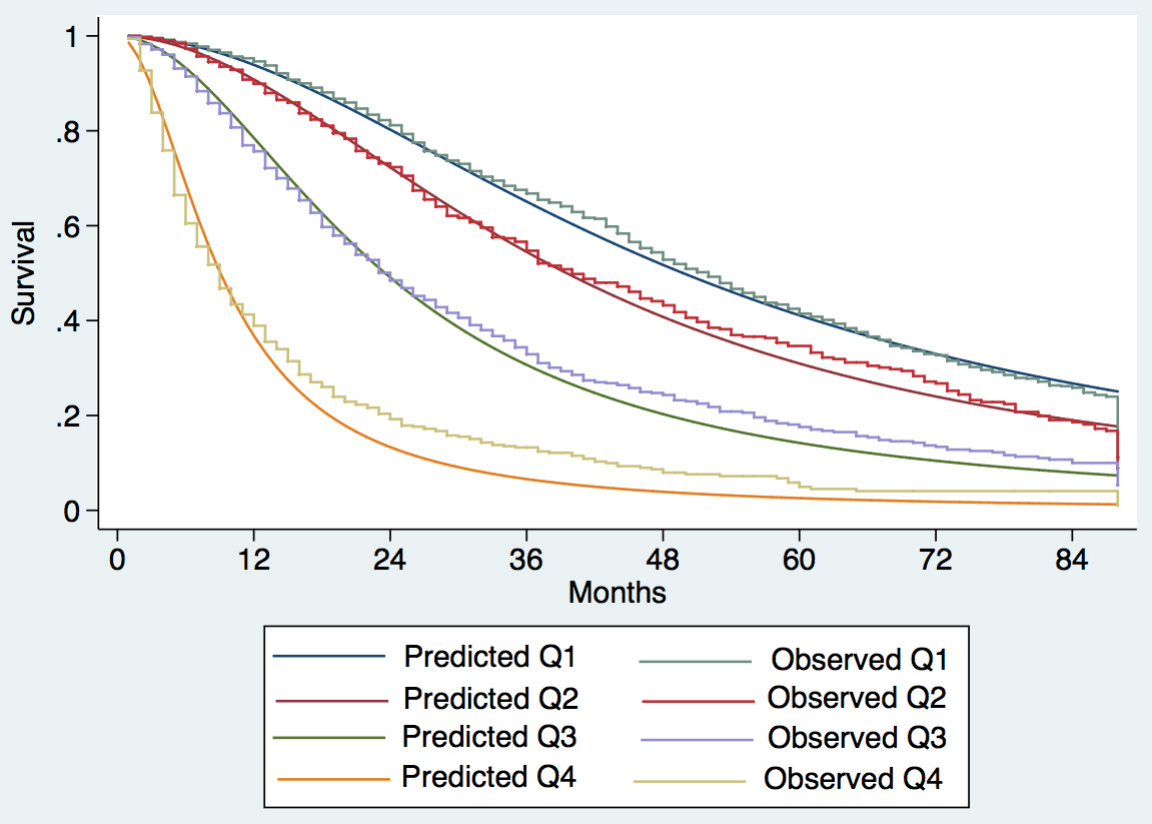
Expected versus observed survival in the training cohort. Patients were divided into quartiles at the 25th, 50th, and 75th percentiles of the risk score. Quartile 1 coincided with ITA.LI.CA score ≤ 1, quartile 2 with score 2–3, quartile 3 with score 4–5, quartile 4 with score >5.

**Fig 2:**
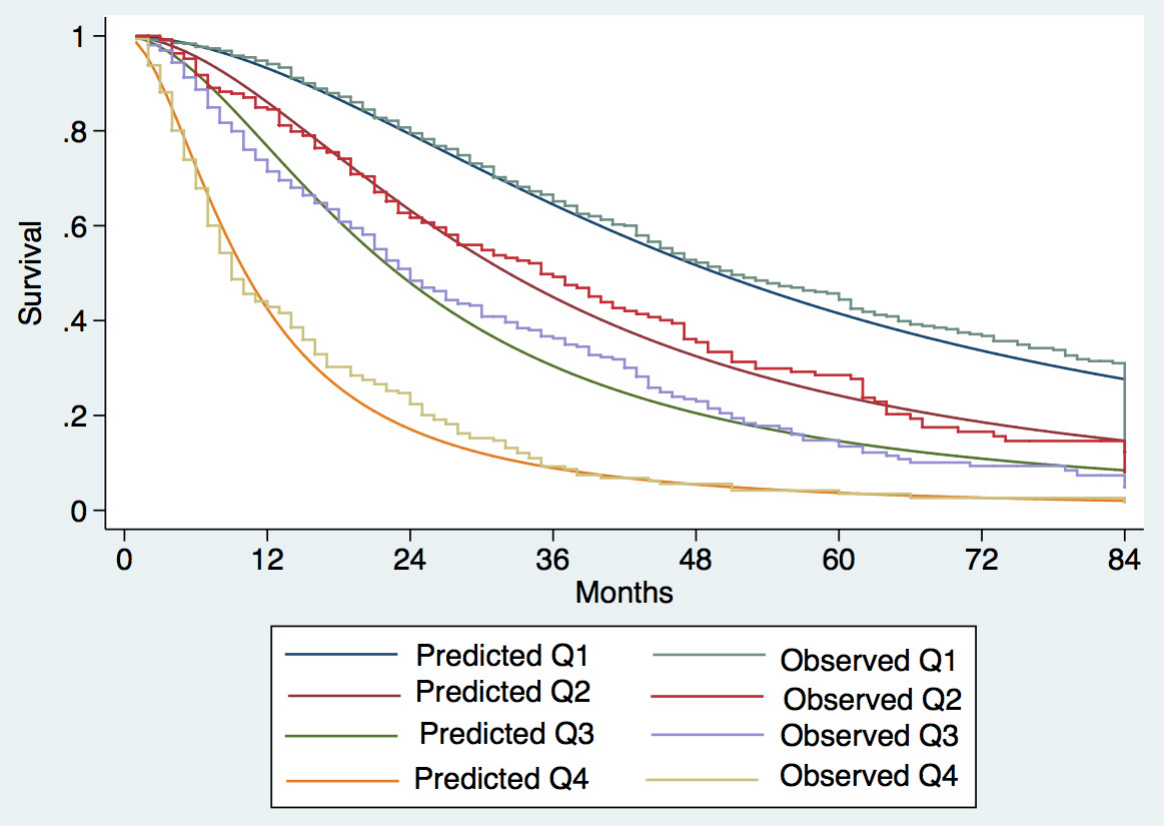
Expected versus observed survival in the internal validation cohort. Patients were divided into quartiles at the 25th, 50th, and 75th percentiles of the risk score. Quartile 1 coincided with ITA.LI.CA score ≤ 1, quartile 2 with score 2–3, quartile 3 with score 4–5, quartile 4 with score > 5.

**Fig 3:**
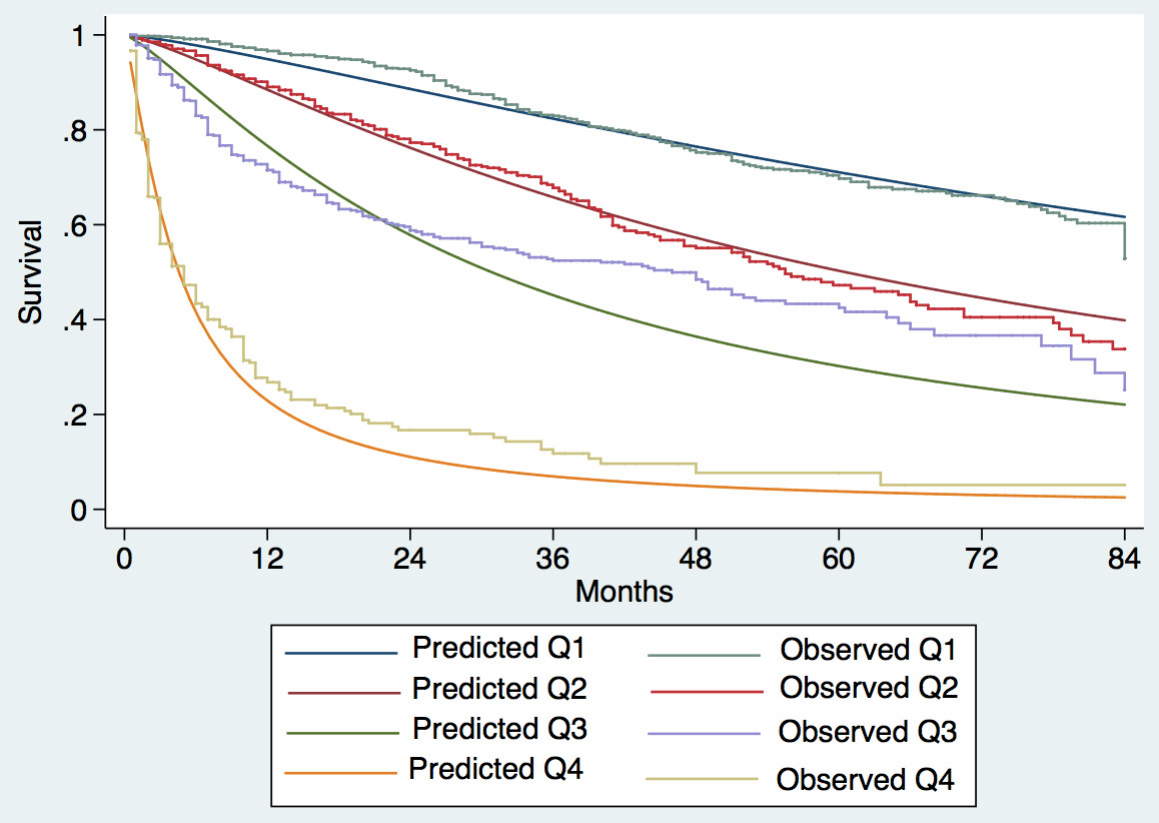
Expected versus observed survival in the external validation cohort. Patients were divided into quartiles at the 25th, 50th, and 75th percentiles of the risk score. Quartile 1 coincided with ITA.LI.CA score ≤ 1, quartile 2 with score 2–3, quartile 3 with score 4–5, quartile 4 with score > 5.

Observed median survival in the training, internal, and external validation cohort (Figs 1–3) was 57, 61, and >129 mo, respectively, in quartile 1; 43, 38, and 75 mo, respectively, in quartile 2; 23, 23, and 56 mo, respectively, in quartile 3; and 9, 8, and 10 mo, respectively, in quartile 4. The calibration was optimal in the ITA.LI.CA training and validation sets (Figs 1 and 2). In the external validation set, calibration was better for quartiles 1 and 2 than for quartiles 3 and 4 (Fig 3).

The ITA.LI.CA score showed the best discriminatory ability and monotonicity of gradients among the most common HCC staging systems (Table 4) in all three study cohorts (training, internal validation, and external validation). In particular, the C index of the ITA.LI.CA score in the internal and external validation cohorts was 0.71 and 0.78, respectively. The ITA.LI.CA score’s prognostic ability was superior to that of BCLC stage (respective C indexes of 0.64 and 0.73), CLIP score (0.68 and 0.75), JIS stage (0.67 and 0.70), MESIAH score (0.69 and 0.77), and HKLC stage (0.68 and 0.75). By using the likelihood ratio test to compare different survival models, the prognostic performance of the ITA.LI.CA system always resulted in significantly better discrimination ability (*p <* 0.001) than the other systems in all three study groups. The superiority of the ITA.LI.CA score was also confirmed after stratification of the analysis for time period (Table 5).

**Table 4:** Discrimination ability of the integrated ITA.LI.CA prognostic system and comparison with other staging systems in the training, internal validation, and external validation cohorts.

HCC Staging System	Training Cohort (*n* = 3,628)				Internal Validation Cohort (*n* = 1,555)				External Validation Cohort (*n* = 2,651)			
	AIC	C Index	Test for Trend χ^2^	LR Test	AIC	C Index	Test for Trend χ^2^	LR Test	AIC	C Index	Test for Trend χ^2^	LR Test
ITA.LI.CA	21,123	0.72	1,090	—	9,154	0.71	486	—	9,133	0.78	1,091	—
CLIP [12]	21,352	0.69	846	265*	9,219	0.68	390	93*	9,241	0.75	912	159*
HKLC [8]	21,408	0.68	539	297*	9,296	0.68	251	97*	9,404	0.75	621	318*
MESIAH [17]	21,516	0.69	654	415*	9,260	0.69	343	131*	9,216	0.77	731	141*
JIS [15]	21,623	0.67	588	508*	9,306	0.67	281	170*	9,691	0.70	428	610*
Modified BCLC [20]	21,727	0.66	474	614*	9,327	0.66	231	197*	9,493	0.75	444	424*
BCLC [6]	21,857	0.65	357	745*	9,379	0.64	169	251*	9,506	0.73	407	412*

The lower the AIC value, the higher the discriminatory ability of the staging system. The higher the C index and the test for trend chi-square, the higher the discriminatory ability and monotonicity of gradients of the staging system. The ITA.LI.CA score was compared with the other systems using the likelihood ratio test.

*p < 0.001.

LR test, likelihood ratio test.

**Table 5:** Discrimination ability of the integrated ITA.LI.CA prognostic system and comparison with other staging systems in the training and internal validation cohorts (*n* = 5,183) stratified based on study period.

Period	HCC Staging System	AIC	C Index	LR Test
**1987–2002 (*n* = 1,902)**	ITA.LI.CA	14,676	0.71	—
	CLIP	14,805	0.68	148.13*
	HKLC	14,861	0.68	195.08*
	MESIAH	14,945	0.67	243.64*
	JIS	14,980	0.66	320.92*
	Modified BCLC	15,048	0.65	390.96*
	BCLC	15,068	0.64	410.87*
**2003–2012 (*n* = 3,281)**	ITA.LI.CA	15,558	0.72	—
	CLIP	15,721	0.69	215.38*
	HKLC	15,728	0.70	179.83*
	MESIAH	15,772	0.69	285.23*
	JIS	15,898	0.68	356.03*
	Modified BCLC	15,952	0.68	411.35*
	BCLC	16,119	0.65	578.49*

The higher the C index, the higher the discriminatory ability and monotonicity of gradients of the staging system. The ITA.LI.CA score was compared with the other systems using the likelihood ratio test.

*p < 0.001.

LR test, likelihood ratio test.

## Discussion

The main purpose of this study is the proposal of a new ITA.LI.CA prognostic system for HCC including both tumor staging, with the potential to be used in the clinical management of HCC patients, and an integrated prognostic score, to predict individual survival. This new prognostic system has a strong prognostic ability in at least one European and one Asian population.

The ITA.LI.CA tumor staging was defined by specific stages (0, A, B1, B2, B3, and C) representing a synthesis of recent data from the literature on HCC prognosis (Table 1) [7,8,23,27]. The use of ITA.LI.CA tumor staging and CPS to develop the prognostic score was also supported by their comparison with other tumor staging and liver function assessment systems described in the literature (S1 Table). In particular, the ITA.LI.CA tumor staging showed a higher discrimination ability than UNOS TNM staging [7] and HKLC staging [8], and CPS had a higher prognostic power than MELD score [28] and the new albumin—bilirubin grade [29].

The definition of some main prognostic subgroups of HCC patients (i.e., tumor stages) may be useful to drive common therapeutic strategies or to design clinical trials [3–5]. However, tumor staging may be inaccurate in determining individual prognosis [10]. For this reason, we integrated the ITA.LI.CA tumor staging with CPS, ECOG PST, and AFP in a multivariable survival model to construct the ITA.LI.CA prognostic system. We investigated the discrimination ability of the ITA.LI.CA score relative to other systems. Of note, our system showed the highest discrimination ability in all study cohorts (Table 4). BCLC staging has been considered by American and European guidelines to be the best system to predict survival in HCC patients [4,5]. However, several studies have shown that this staging system has several limitations, mainly related to the large heterogeneity of BCLC stage B and C patients [23], the controversial prognostic role of ECOG PST [20] (which can be influenced by liver function, cancer symptoms, or both), and the lack of statistical weighting of different factors such as tumor status, liver function variables, and ECOG PST [8,10]. In the current study, BCLC staging had the lowest prognostic power among the different prognostic systems, while the ITA.LI.CA score had the highest prognostic power (Table 4).

Therapeutic choice is probably the most important prognostic factor for HCC patients [3]. However, none of the proposed HCC prognostic systems [6–17] include this variable for two main reasons. From a clinical point of view, treatment choice is extremely difficult to predict, especially in HCC patients, for whom many therapies are available and many variables influence treatment decision. From a statistical point of view, there are many interactions between therapy and other prognostic variables (i.e., tumor size and CPS influence simultaneously treatment decision and survival). A potential solution to this problem is to not include treatment as a prognostic variable, but rather consider treatment decision as an outcome variable, proposing a treatment scheme linked to the staging system (i.e., BCLC or HKLC algorithm) [3–6,8]. Treatment algorithms, such as the BCLC and HKLC ones, however, are by nature rigid tools, giving only one treatment option for each stage or sub-stage, and intrinsically not open to treatment alternatives and evolution [23]. This difficulty explains why, in everyday clinical practice, HCC guidelines are usually disregarded [30–35].

Based on these considerations, we deliberately did not propose a treatment algorithm in the present study. Although this study was designed with a prognostic intent, the variables included in the ITA.LI.CA prognostic system have an established impact on treatment decision, as recently underlined by the position paper of the Italian Association for the Study of the Liver [28]: (a) size and number of nodules and vascular invasion (used to determine tumor stage 0, A, B1, B2, B3, C), (b) the liver function and patient-related variables CPS and ECOG PST (the ITA.LI.CA functional score), and (c) AFP level. In particular, the ITA.LI.CA tumor staging preserves the basic staging system of very early (0), early (A), intermediate (B1, B2, and B3), and advanced (C) stages, since staging according to this system is the main criterion for treatment decisions in current guidelines. CPS and ECOG PST (composing the ITA.LI.CA functional score) and AFP level are well known variables used to evaluate treatment feasibility and aggressiveness within each tumor stage [3–5,28].

For clinical use, the ITA.LI.CA prognostic system can be synthetized in a single formula, TS_FA_, where TS is the tumor stage, F is point value of the ITA.LI.CA functional score, and A is the AFP point value. For example, the formula A_22_ indicates a patient with a stage A HCC, with an ITA.LI.CA functional score of two points (CPS of 6–7 and PST of 1–2 or CPS of 8–9 and PST of 0), and with two points for AFP (>1,000 μg/l); the formula C_00_ indicates a stage C HCC, with a CPS of 5 and a PST of 0 and with AFP ≤ 1,000. Considering that one point is assigned to each tumor stage (Table 3, from 0 to 5 for stages from 0 to C), this simplified, user-friendly formula can synthetize all ITA.LI.CA system components and the prognostic score, but also provide an accurate clinical description of each HCC patient with the potential to be used for deciding patient treatment or designing clinical trials.

Unlike other HCC prognostic scores not linked to treatment recommendations [11–17], the ITA.LI.CA integrated prognostic system is compatible both with current guidelines [3–5,28] and with a more modern management of HCC patients. Recent evidence, for example, supports expanded use of surgery as first-line therapy regardless of tumor status, provided that it is technically and oncologically feasible [30–33], and the use of percutaneous ablation and intra-arterial therapies in conjunction for early and intermediate HCCs [34,36].

This study has several potential limitations, however. The main limitation of this study is its retrospective nature. A prospective trial would be the ideal setting to validate the ITA.LI.CA prognostic system. On the other hand, the high number of enrolled patients, the strong methodology (internal and external validation), and the heterogeneity in treatment allocation during the study period gave us the opportunity to compare different prognostic systems. Such a comparison would be really difficult in a prospective trial in both ethical and statistical (sample size) terms. Moreover, it has to be underlined that all main prognostic systems currently used for HCC patients [6–17] were developed using retrospective series based on cohorts significantly smaller than that used in the present study.

A second limitation concerns the intrinsically significant differences between the Taiwanese and Italian groups (Table 2), particularly in cancer etiology, laboratory data, tumor stage distribution, and, most importantly, treatment choices. In particular, we would underline the crucial issue of differences in etiology among European and Asian countries [33,36,37]. Another important difference between the Italian and Taiwan cohorts is the different enrollment periods. All these differences could potentially introduce selection biases. In this study, we performed specific subgroup analyses showing that the prognostic power of the ITA.LI.CA prognostic system was not influenced by enrollment period (Table 5).

The differences between the Taiwanese and Italian groups probably explain why the survival of Taiwanese patients was significantly greater than that of Italian patients with the same ITA.LI.CA score (Figs 1–3 and S2 Fig). On the other hand, the ITA.LI.CA system performed as the best prognostic score also in the Taiwanese patients (Tables 4 and 5). Thus, although the Italian and Taiwanese groups differed in their survival estimations, this study demonstrated a broad applicability of the proposed system both in European and Asian populations.

In conclusion, the ITA.LI.CA prognostic system demonstrated the best ability of the compared systems to predict the survival of Italian and Taiwanese patients. Moreover, it allows a simple but accurate clinical description of each HCC patient, with the potential to be used for deciding treatment or designing clinical trials.

## Supporting Information

S1 TRIPOD checklist Prediction model development and validation. (DOCX)

S1 Fig Observed versus predicted survival curves according to main ITA.LI.CA tumor stages in the training set (0 versus A versus B versus C tumor stages): survival curves predicted using a Cox model. (TIF)

S2 Fig Observed versus predicted survival curves according to main ITA.LI.CA tumor stages in the training set (0 versus A versus B versus C tumor stages): survival curves predicted using a log-logistic parametric model. (TIF)

S3 Fig Overall Kaplan—Meier survival curve of the training and internal validation groups from Italy (*n* = 5,183) versus the external validation group from Taiwan (*n* = 2,651). Log-rank test, *p <* 0.001. (TIF)

S1 Table Discrimination ability of the ITA.LI.CA tumor staging and CPS covariates, and their comparison with other tumor staging and liver function systems in the training, internal validation, and external validation cohorts. (DOCX)

S2 Table Distribution of patients with different points in the ITA.LI.CA score and relative observed median survival rates. Log-rank test, *p* < 0.001.
(DOCX)

S1 Text Details about patient data collected for this study. (DOCX)

S2 Text Development of the ITA.LI.CA prognostic system. (DOCX)

S3 Text Validation of the ITA.LI.CA prognostic system. (DOCX)

S4 Text References used in the Supporting Information files. (DOCX)

## Abbreviations

AFP: alpha-fetoprotein
AIC: Akaike information criterion
BLCL: Barcelona Clinic Liver Cancer
C index: concordance index
CLIP: Cancer of the Liver Italian Program
CPS: Child—Pugh score
ECOG: Eastern Cooperative Oncology Group
HCC: hepatocellular carcinoma
HKLC: Hong Kong Liver Cancer
ITA.LI.CA: Italian Liver Cancer
JIS: Japan Integrated Staging
MELD: Model for End-Stage Liver Disease
MESIAH: Model to Estimate Survival in Ambulatory HCC Patients
PST: performance status test
TNM: tumor node metastases
UNOS: United Network for Organ Sharing
