# Supplementary figures and images for: Development and Validation of a New Prognostic System for Patients with Hepatocellular Carcinoma
Source: PLoS Med. 2016 Apr 26;13(4):e1002006. doi: 10.1371/journal.pmed.1002006 (PMC4846017; doi:10.1371/journal.pmed.1002006)

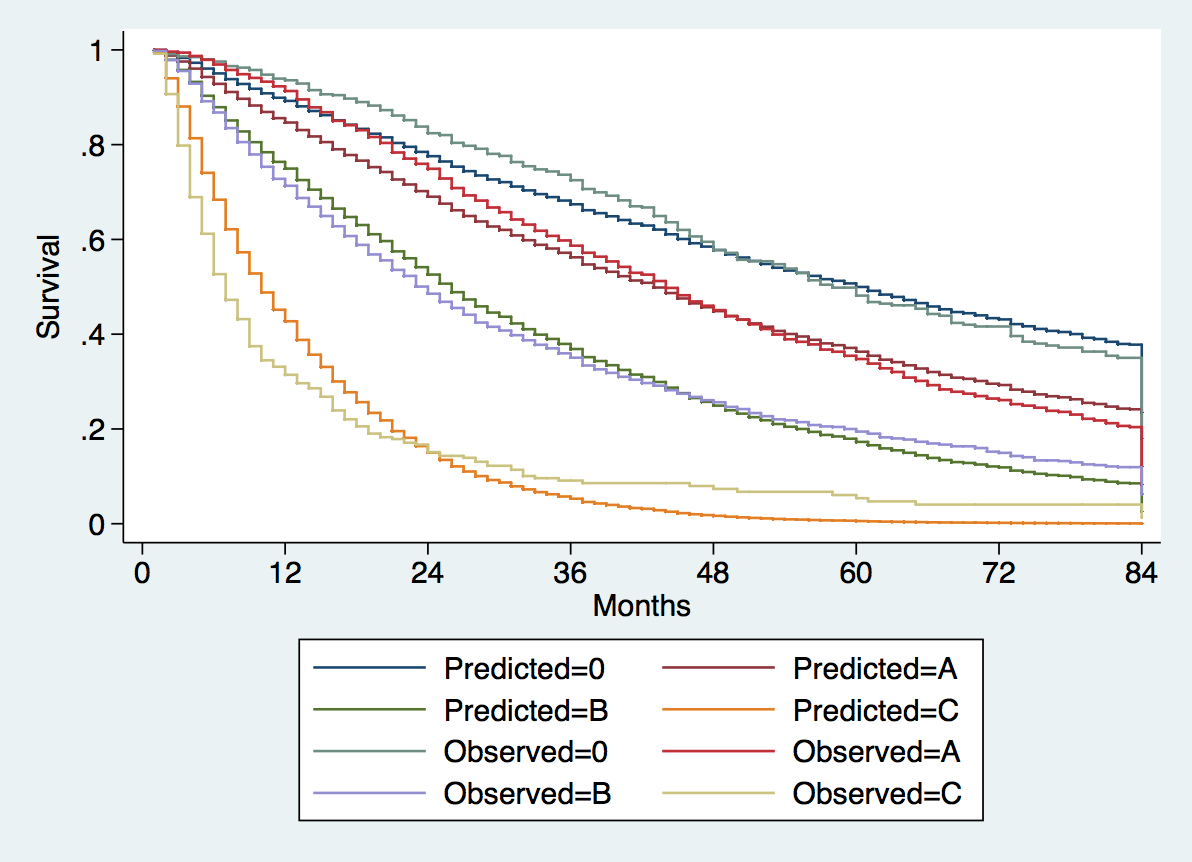

Supplement: S1 Fig — (TIF) [file pmed.1002006.s002.tif]

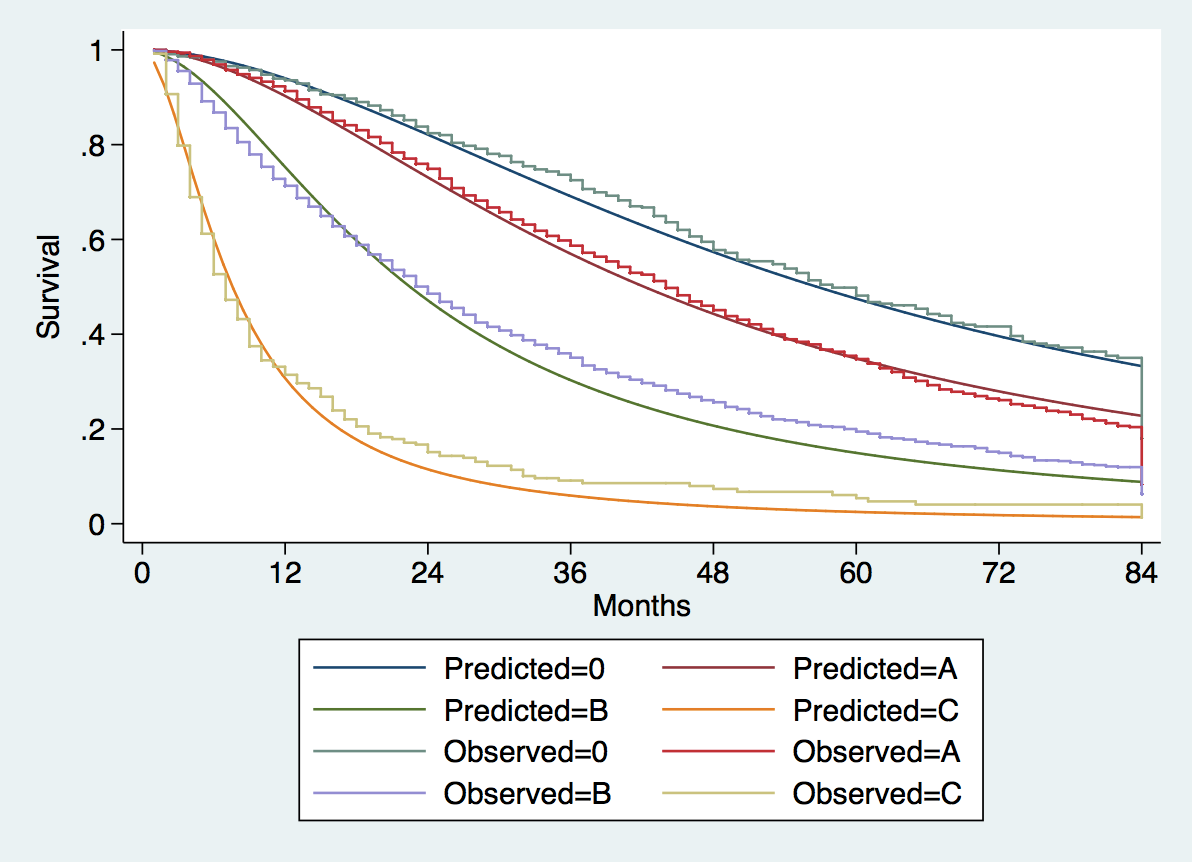

Supplement: S2 Fig — (TIF) [file pmed.1002006.s003.tif]

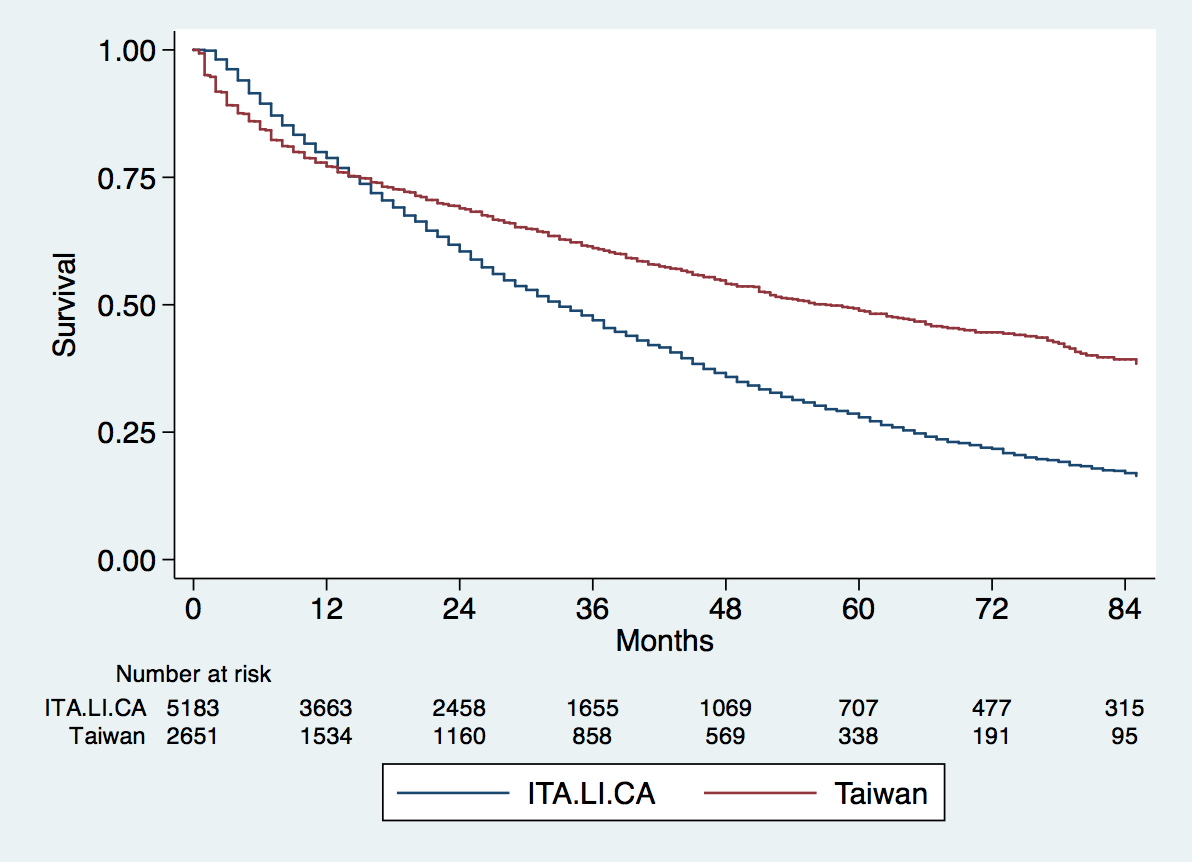

Supplement: S3 Fig — (TIF) [file pmed.1002006.s004.tif]
