# Supplementary material for: Development and Validation of a New Prognostic System for Patients with Hepatocellular Carcinoma
Source: PLoS Med. 2016 Apr 26;13(4):e1002006. doi: 10.1371/journal.pmed.1002006 (PMC4846017; doi:10.1371/journal.pmed.1002006)
Supplement: S1 Table — (DOCX) [file pmed.1002006.s005.docx]

**S1 Table.** *Discrimination ability of the ITA.LI.CA tumor staging and CPS covariates, and their comparison with other tumor staging and liver function systems in the training, internal validation, and external validation cohorts.*

|  | **Training** **cohort**  **(n=3,628)** | | | **Internal validation cohort (n=1,555)** | | | **External validation cohort** **(n=2,651)** | | |
| --- | --- | --- | --- | --- | --- | --- | --- | --- | --- |
| **Tumor staging systems** | **AIC** | **C Index** | **lrtest, p value** | **AIC** | **C Index** | **lrtest, p value** | **AIC** | **C Index** | **lrtest, p value** |
| ITA.LI.CA | 21,594 | 0.68 | - | 9,274 | 0.68 | - | 9,743 | 0.70 | - |
| TNM (UNOS)^10^ | 21,727 | 0.67 | 100.01  <.0001 | 9,318 | 0.66 | 46.17  <.0001 | 9,849 | 0.66 | 110.11  <.0001 |
| TNM (JAPAN)^11^ | 21,935 | 0.64 | 307.28  <.0001 | 9,414 | 0.63 | 142.46  <.0001 | 10,072 | 0.59 | 335.31  <.0001 |
| Hong Kong Tumor stage^6^ | 21,643 | 0.66 | 15.51  .0004 | 9,296 | 0.65 | 27.42  <.0001 | 9,771 | 0.68 | 34.58  <.0001 |
| **Liver function systems** |  |  |  |  |  |  |  |  |  |
| CPS^4^ | 22,076 | 0.63 | - | 9,428 | 0.63 | - | 9,594 | 0.72 | - |
| MELD score^12^ | 22,240 | 0.59 | 174.95  <.0001 | 9,489 | 0.61 | 72.76  <.0001 | 9,851 | 0.67 | 301.45  <.0001 |
| MELD sodium score^13^ | 22,166 | 0.61 | 100.85  <.0001 | 9,451 | 0.62 | 33.08  <.0001 | 9,724 | 0.70 | 177.13  <.0001 |
| ALBI grade^14^ | 22,162 | 0.62 | 98.25  <.0001 | 9,449 | 0.61 | 51.00  <.0001 | 9,669 | 0.72 | 122.86  <.0001 |

In each column have been reported the Akaike Information Criterion (AIC) as first value, and the C-index as second value, The higher the c-index and the test for trend chi-square, the higher the discriminatory ability and monotonicity of gradients of the staging system. In addition, in each column the ITA.LI.CA score was compared with other systems by using the likelihood ratio test.

Abbreviations: ITA.LI.CA, Italian Liver Cancer; TNM, tumor node metastasis; CPS, Child Pugh score; MELD, model for end-stage liver disease; ALBI, albumin-bilirubin.
