# Supplementary material for: Development and Validation of a New Prognostic System for Patients with Hepatocellular Carcinoma
Source: PLoS Med. 2016 Apr 26;13(4):e1002006. doi: 10.1371/journal.pmed.1002006 (PMC4846017; doi:10.1371/journal.pmed.1002006)
Supplement: S2 Table — Log-rank test, p < 0.001. (DOCX) [file pmed.1002006.s006.docx]

**S2 Table.** *Distribution of patients in different points of the score and relative observed median survivals.*

*Log Rank test, p < .0001*

| **Score** | **Number of patients (%)** | **Observed Median survival (months)** | **Lower 95%** | **Higher 95%** |
| --- | --- | --- | --- | --- |
| 0 | 273 (5.3) | 73 | 60 | 84 |
| 1 | 895 (17.3) | 54 | 50 | 60 |
| 2 | 946 (18.3) | 44 | 40 | 46 |
| 3 | 924 (17.8) | 36 | 34 | 40 |
| 4 | 671 (12.9) | 26 | 24 | 29 |
| 5 | 506 (9.8) | 18 | 16 | 20 |
| 6 | 334 (6.4) | 13 | 11 | 15 |
| 7 | 247 (4.8) | 8 | 7 | 10 |
| 8 | 162 (3.1) | 6 | 5 | 8 |
| 9 | 111 (2.1) | 5 | 4 | 7 |
| 10 | 67 (1.3) | 4 | 3 | 6 |
| 11 | 30 (0.6) | 4 | 3 | 6 |
| 12 | 10 (0.2) | 3 | 0 | 6 |
| 13 | 7 (0.1) | 2 | 1 | 3 |
| Combined | 5183 | 32 | 31 | 34 |
