# Supplementary material for: Development and Validation of a New Prognostic System for Patients with Hepatocellular Carcinoma
Source: PLoS Med. 2016 Apr 26;13(4):e1002006. doi: 10.1371/journal.pmed.1002006 (PMC4846017; doi:10.1371/journal.pmed.1002006)
Supplement: S1 Text — (DOCX) [file pmed.1002006.s007.docx]

**Variables**

Only patients with diagnosis of HCC confirmed either by histology or cytology, or by typical radiologic appearance, were included in the study group.^1, 2^ Clinical and treatment-related variables, such as age, sex, etiology of underlying liver disease, presence of ascites and hepatic encephalopathy, main serological parameters (total bilirubin, creatinine, prothrombin time and/or INR, α‑fetoprotein, albumin, sodium), Child-Pugh score (CPS) ^3,4^, tumor radiological characteristics (number and size of lesions), Eastern Cooperative Oncology Group performance status (ECOG PST) ^5^, and main treatment strategy (hepatic resection, percutaneous ablation, intra-arterial therapies, or other options) were recorded. ECOG PST was prospectively assessed by clinicians who participated to the ITA.LI.CA database. ^1^ Tumor number and size, major vascular invasion and patterns of metastatic diffusion were assessed by computer tomography (CT) or magnetic resonance imaging (MRI). Specifically, vascular invasion was classified in intra- and extra-hepatic, following the criteria of the Hong Kong Liver Cancer (HKLC) Staging System^6^. Intrahepatic vascular invasion included intrahepatic portal vein branch, left or right portal vein invasion, and main hepatic vein invasion; extrahepatic vascular invasion includes main portal vein and inferior vena cava invasion.

In the database from Taipei the variable macroscopic vascular invasion was not distinguished into intra- and extra-hepatic. Macroscopic vascular invasion in patients from Taiwan was therefore considered as extra-hepatic in all staging systems.

We considered seven main therapeutic subgroups: hepatic resection, transplantation, ablation (percutaneous alcoholization, radiofrequency and microwave), intra-arterial therapies (IAT), sorafenib, other palliative treatment (systemic chemotherapy) and best supportive care (BSC).

Firstly, we selected all patients undergoing liver transplantation (transplantation group, n=106) and we followed them from the time of surgery onwards. These patients were considered in the transplantation group even if they underwent other HCC therapies. Secondly, we selected all patients undergoing liver resection (resection group, n=554) and we followed them from the time of resection onwards. Then, we selected patients undergoing at least one ablation procedure (ablation group, n= 1,519) and we followed them from the time of first ablation onwards, independently from other non-surgical treatment received during their follow-up. Similarly, we defined other treatment groups in the following order: the IAT group (n=1,371), the Sorafenib group (n=160), the other systemic therapy group (n=437), and the BSC group (n=1,036).

The same criteria have been used to define treatment subgroups in the external validation cohort.

Tumor staging was only based on radiology for all ITA.LI.CA patients (surgical and not surgical).
