# Supplementary material for: Development and Validation of a New Prognostic System for Patients with Hepatocellular Carcinoma
Source: PLoS Med. 2016 Apr 26;13(4):e1002006. doi: 10.1371/journal.pmed.1002006 (PMC4846017; doi:10.1371/journal.pmed.1002006)
Supplement: S2 Text — (DOCX) [file pmed.1002006.s008.docx]

**Development of the ITA.LI.CA prognostic system**

To find the best model to use in the survival analysis, we used both a statistical test, the Schoenfeld residual test, and a graphical method, observed vs. predicted survival curves plot, to check if model covariates followed the proportional hazard assumption. We found that liver tumor status variable (S1 Figure) did not follow this assumption.

Since the aim of this study was to develop a model able to give precise survival estimation, we decided to use an accelerated failure time parametric model. Among the different models available (gamma, log-logistic, lognormal), we selected the log-logistic model since it showed the lowest value of Akaike Information Criterion (AIC). ^7^ As shown in the supplementary S2 figure, the log-logistic model well predicted survival according tumor status variable.

The prognostic discrimination ability of the ITA.LI.CA tumor staging was compared, using the Akaike information criterion (AIC),[^7^](#_ENREF_26)^, 8^ and the Concordance (C)-index, to that of other tumor staging systems, the UNOS TNM^9^, the Japan TNM^10^, and Hong Kong tumor stage[^6^](#_ENREF_27) (Table S1). The lower the AIC value and the higher the c-index, the higher the discriminatory ability of the staging system. Similarly, the discrimination ability of CPS was compared to that of other liver function systems, the model for end-stage liver disease (MELD) score^11^, the MELD sodium score^12^, and the albumin-bilirubin (ALBI) grade^13^ (Table S1). As shown in Table S1, the ITA.LI.CA tumor staging and the CPS score proved the best discrimination ability in all the study groups. To measure if the performance of the ITA.LI.CA tumor staging and of CPS was significantly better than that of other systems we used the likelihood ratio test.

ITA.LI.CA tumor staging, and CPS score were thus used together with ECOG PS and AFP to construct the ITA.LI.CA prognostic score in the training cohort (n=3,628).

To weight the relative contribution of each covariate to survival prediction, these variables were included in a multivariate log-logistic survival model.

A prognostic score was generated using the independent variables weighed according to the estimated regression coefficient of the final model. The reference category of each prognostic factor was assigned a value of zero. A simplified version was derived from the original model by linear transformation of the estimates (estimates * 3.5, rounded).
