# Supplementary material for: Development and Validation of a New Prognostic System for Patients with Hepatocellular Carcinoma
Source: PLoS Med. 2016 Apr 26;13(4):e1002006. doi: 10.1371/journal.pmed.1002006 (PMC4846017; doi:10.1371/journal.pmed.1002006)
Supplement: S3 Text — (DOCX) [file pmed.1002006.s009.docx]

**Validation of the ITA.LI.CA prognostic system**

Calibration, discriminatory ability, and monotonicity of gradients were evaluated to measure the prognostic ability of the ITA.LI.CA prognostic score.[^8^](#_ENREF_25) In testing calibration of the model prediction in the training, internal and external validation cohorts, patients were divided into four groups at the 25^th^, 50th and 75th percentiles of the risk score. The calibration was defined as the graphical agreement between observed outcomes and predictions.

The discriminatory ability of staging systems was described using the Harrel’s C index, and the AIC[^7^](#_ENREF_26). Considering prognostic scores as ordinary variables, the monotonicity of gradients in mortality rates were quantified using the test for trend of survivor functions. The results obtained for the ITA.LI.CA prognostic score in terms of discriminatory ability and monotonicity of gradients were then compared with those for the BCLC staging system, modified BCLC, CLIP, HKLC, MESIAH, JIS. ^5,6, 14-17^

Due to the long enrollment period (from 1987 to 2012), a subgroup analysis for time period was also performed to evaluate and overcome potential time related biases.
