# Supplementary material for: Development and Validation of a New Prognostic System for Patients with Hepatocellular Carcinoma
Source: PLoS Med. 2016 Apr 26;13(4):e1002006. doi: 10.1371/journal.pmed.1002006 (PMC4846017; doi:10.1371/journal.pmed.1002006)
Supplement: S4 Text — (DOCX) [file pmed.1002006.s010.docx]

**References used in the supporting information files**

1. European Association For The Study Of The L, European Organisation For R, Treatment Of C. EASL-EORTC clinical practice guidelines: management of hepatocellular carcinoma. *Journal of hepatology* 2012;**56**(4): 908-943.
2. Italian Association for the Study of the L, Panel AE, Committee AC, Bolondi L, Cillo U, Colombo M, Craxi A, Farinati F, Giannini EG, Golfieri R, Levrero M, Pinna AD, Piscaglia F, Raimondo G, Trevisani F, Bruno R, Caraceni P, Ciancio A, Coco B, Fraquelli M, Rendina M, Squadrito G, Toniutto P. Position paper of the Italian Association for the Study of the Liver (AISF): the multidisciplinary clinical approach to hepatocellular carcinoma. *Digestive and liver disease : official journal of the Italian Society of Gastroenterology and the Italian Association for the Study of the Liver* 2013;**45**(9): 712-723.
3. Child CG, Turcotte JG. Surgery and portal hypertension. *Major problems in clinical surgery* 1964;**1**: 1-85.
4. Pugh RN, Murray-Lyon IM, Dawson JL, Pietroni MC, Williams R.
5. Hsu CY, Lee YH, Hsia CY, Huang YH, Su CW, Lin HC, Lee RC, Chiou YY, Lee FY, Huo TI. [Performance status in patients with hepatocellular carcinoma: determinants, prognostic impact, and ability to improve the Barcelona Clinic Liver Cancer system.](http://www.ncbi.nlm.nih.gov/pubmed/22806819) Hepatology. 2013 Jan;57(1):112-9. doi: 10.1002/hep.25950.
6. Yau T, Tang VY, Yao TJ, Fan ST, Lo CM, Poon RT. Development of Hong Kong Liver Cancer staging system with treatment stratification for patients with hepatocellular carcinoma. *Gastroenterology* 2014;**146**(7): 1691-1700
7. M S. Akaike’s criteria. In: Armitage P, Colton T, eds Encyclopedia of Biostatistics. Chichester: Wiley; 1998. p. 123-124.
8. Steyerberg EW, Vickers AJ, Cook NR, Gerds T, Gonen M, Obuchowski N, Pencina MJ, Kattan MW. Assessing the performance of prediction models: a framework for traditional and novel measures. *Epidemiology* 2010;**21**(1): 128-138.
9. UNOS/OPTN policy. <http://optn.transplant.hrsa.gov/SharedContentDocuments/2011-11_Policy_Notice.pdf>. [March 5 2015].
10. Minagawa M, Ikai I, Matsuyama Y, Yamaoka Y, Makuuchi M. [Staging of hepatocellular carcinoma: assessment of the Japanese TNM and AJCC/UICC TNM systems in a cohort of 13,772 patients in Japan.](http://www.ncbi.nlm.nih.gov/pubmed/17522517)

Ann Surg 2007; 245: 909-922.

1. Freeman RB, Edwards EB, Harper AM. Waiting list removal rates among patients with chronic and malignant liver diseases. Am J Transplant 2006 Jun;6: 1416–1421.
2. Kim WR, Biggins SW, Kremers WK, et al. Hyponatremia and mortality among patients on the liver-transplant waiting list. N Engl J Med 2008; 359: 1018–1026.
3. Johnson PJ, Berhane S, Kagebayashi C, et al.  Assessment of Liver Function in Patients With Hepatocellular Carcinoma: A New Evidence-Based Approach—The ALBI Grade. J Clin Oncol 2015; 20; 33: 550-558.
4. A new prognostic system for hepatocellular carcinoma: a retrospective study of 435 patients: the Cancer of the Liver Italian Program (CLIP) investigators. *Hepatology* 1998;**28**(3): 751-755.
5. Kudo M, Chung H, Osaki Y. Prognostic staging system for hepatocellular carcinoma (CLIP score): its value and limitations, and a proposal for a new staging system, the Japan Integrated Staging Score (JIS score). *Journal of gastroenterology* 2003;**38**(3): 207-215.
6. Llovet JM, Bru C, Bruix J. Prognosis of hepatocellular carcinoma: the BCLC staging classification. *Seminars in liver disease* 1999;**19**(3): 329-338.
7. Yang JD, Kim WR, Park KW, Chaiteerakij R, Kim B, Sanderson SO, Larson JJ, Pedersen RA, Therneau TM, Gores GJ, Roberts LR, Park JW. Model to estimate survival in ambulatory patients with hepatocellular carcinoma. *Hepatology* 2012;**56**(2): 614-621.
8. Yin L, Li H, Li AJ, Lau WY, Pan ZY, Lai EC, Wu MC, Zhou WP. [Partial hepatectomy vs. transcatheter arterial chemoembolization for resectable multiple hepatocellular carcinoma beyond Milan Criteria: a RCT.](http://www.ncbi.nlm.nih.gov/pubmed/24650695)

J Hepatol. 2014; **61**(1): 82-88.

1. Liu H, Wang ZG, Fu SY, Li AJ, Pan ZY, Zhou WP, Lau WY, Wu MC. [Randomized clinical trial of chemoembolization plus radiofrequency ablation versus partial hepatectomy for hepatocellular carcinoma within the Milan criteria.](http://www.ncbi.nlm.nih.gov/pubmed/26780107) Br J Surg. 2016 Jan 18. doi: 10.1002/bjs.10061.
2. Torzilli G, Belghiti J, Kokudo N, Takayama T, Capussotti L, Nuzzo G, et al. A snapshot of the effective indications and results of surgery for hepatocellular carcinoma in tertiary referral centers: is it adherent to the EASL/AASLD recommendations?: an observational study of the HCC East-West study group. *Annals of surgery* 2013;**257**(5): 929-937.
3. Roayaie S, Jibara G, Tabrizian P, Park JW, Yang J, Yan L, et al. The role of hepatic resection in the treatment of hepatocellular cancer. Hepatology. 2015; 62: 440-451.
4. Vitale A, Burra P, Frigo AC, Trevisani F, Farinati F, Spolverato G, et al. Italian Liver Cancer g. Survival benefit of liver resection for patients with hepatocellular carcinoma across different Barcelona Clinic Liver Cancer stages: a multicentre study. *Journal of hepatology* 2015;**62**(3): 617-624.
5. [Zhong JH](http://www.ncbi.nlm.nih.gov/pubmed/?term=Zhong%20JH%5BAuthor%5D&cauthor=true&cauthor_uid=24096763)1, [Ke Y](http://www.ncbi.nlm.nih.gov/pubmed/?term=Ke%20Y%5BAuthor%5D&cauthor=true&cauthor_uid=24096763), [Gong WF](http://www.ncbi.nlm.nih.gov/pubmed/?term=Gong%20WF%5BAuthor%5D&cauthor=true&cauthor_uid=24096763), [Xiang BD](http://www.ncbi.nlm.nih.gov/pubmed/?term=Xiang%20BD%5BAuthor%5D&cauthor=true&cauthor_uid=24096763), [Ma L](http://www.ncbi.nlm.nih.gov/pubmed/?term=Ma%20L%5BAuthor%5D&cauthor=true&cauthor_uid=24096763), [Ye XP](http://www.ncbi.nlm.nih.gov/pubmed/?term=Ye%20XP%5BAuthor%5D&cauthor=true&cauthor_uid=24096763), [Peng T](http://www.ncbi.nlm.nih.gov/pubmed/?term=Peng%20T%5BAuthor%5D&cauthor=true&cauthor_uid=24096763), et al. Hepatic resection associated with good survival for selected patients with intermediate and advanced-stage hepatocellular carcinoma. Ann Surg 2014; 260: 329-340.
6. Wang Y, Luo Q, Li Y, Deng S, Wei S, Li X. [Radiofrequency ablation versus hepatic resection for small hepatocellular carcinomas: a meta-analysis of randomized and nonrandomized controlled trials.](http://www.ncbi.nlm.nih.gov/pubmed/24404166) PLoS One. 2014; 9(1): e84484.
7. Cucchetti A, Piscaglia F, Cescon M, et al. [Cost-effectiveness of hepatic resection versus percutaneous radiofrequency ablation for early hepatocellular carcinoma.](http://www.ncbi.nlm.nih.gov/pubmed/23603669) J Hepatol. 2013; 59: 300-307.
8. Ito T, Tanaka S, Iwai S, et al. [Outcomes of laparoscopic hepatic resection versus percutaneous radiofrequency ablation for hepatocellular carcinoma located at the liver surface: A case-control study with propensity score matching.](http://www.ncbi.nlm.nih.gov/pubmed/26386248) Hepatol Res. 2015 Sep 19. doi: 10.1111/hepr.12592.
9. Cucchetti A, Cescon M, Golfieri R, Piscaglia F, Renzulli M, Neri F, Cappelli A, Mazzotti F, Mosconi C, Colecchia A, Ercolani G, Pinna AD. Hepatic venous pressure gradient in the preoperative assessment of patients with resectable hepatocellular carcinoma. J Hepatol 2016; 64: 79-86.
10. Cescon M, Cucchetti A, Grazi GL, et al. [Indication of the extent of hepatectomy for hepatocellular carcinoma on cirrhosis by a simple algorithm based on preoperative variables.](http://www.ncbi.nlm.nih.gov/pubmed/19153326) Arch Surg. 2009; 144: 57-63.
11. Peng ZW, Zhang YJ, Chen MS, Xu L, Liang HH, Lin XJ, et al. [Radiofrequency ablation with or without transcatheter arterial chemoembolization in the treatment of hepatocellular carcinoma: a prospective randomized trial.](http://www.ncbi.nlm.nih.gov/pubmed/23269991) J Clin Oncol. 2013; 31: 426-32.
12. Wang Y, Deng T, Zeng L, Chen W. [Efficacy and safety of radiofrequency ablation and transcatheter arterial chemoembolization for treatment of hepatocellular carcinoma: A meta-analysis.](http://www.ncbi.nlm.nih.gov/pubmed/26265000) Hepatol Res. 2015 Aug 11. doi: 10.1111/hepr.12568.
13. Fuks D, Dokmak S, Paradis V, et al. [Benefit of initial resection of hepatocellular carcinoma followed by transplantation in case of recurrence: an intention-to-treat analysis.](http://www.ncbi.nlm.nih.gov/pubmed/21932387) Hepatology. 2012 Jan;55(1):132-40. doi: 10.1002/hep.24680. PMID: 21932387
14. Ferrer-Fàbrega J, Forner A, Liccioni A, et al. [Prospective validation of "ab initio" liver transplantation in hepatocellular carcinoma upon detection of risk factors for recurrence after resection.](http://www.ncbi.nlm.nih.gov/pubmed/26567038) Hepatology. 2015 Nov 13. doi: 10.1002/hep.28339. [Epub ahead of print]
15. N’Kontchou G, Aout M, Laurent A, et al. Survival after radiofrequency ablation and salvage transplantation in patients with hepatocellular carcinoma and Child-Pugh A cirrhosis. J Hepatol 2012; 56: 160-166
16. Mehta N, Dodge JL, Fidelman N, Roberts JP, Yao FY. Intention-to-treat Outcome of T1 Hepatocellular Carcinoma Using the Approach of “Wait and not Ablate” Until Meeting T2 Criteria or Liver Transplant Listing. Hepatology 2013; 58 Suppl 1: 212A [DOI: 10.1002/hep.26792]
17. Cillo U, Vitale A, Volk ML, et al: The survival benefit of liver transplantation in hepatocellular carcinoma patients. Digestive and Liver Disease 2010, 42(9):642-649.
18. Vitale A, Cucchetti A, Qiao GL, et al. Is resectable hepatocellular carcinoma a contraindication to liver transplantation? A novel decision model based on "number of patients needed to transplant" as measure of transplant benefit. Journal of Hepatology 2014, 60(6):1165-1171.
19. Vitale A HT, Cucchetti A, Lee YH, et al. The survival benefit of liver transplantation versus resection for hepatocellular carcinoma: impact of MELD score. Ann Surg Oncol. 2015;22(6):1901-749.
20. Berry K, Ioannou GN. Are patients with Child's A cirrhosis and hepatocellular carcinoma appropriate candidates for liver transplantation? American Journal of Transplantation: official journal of the American Society of Transplantation and the American Society of Transplant Surgeons 2012, 12(3):706-717.
21. Vitale A, Morales RR, Zanus G, et al. Barcelona Clinic Liver Cancer staging and transplant survival benefit for patients with hepatocellular carcinoma: a multicentre, cohort study. The Lancet Oncology 2011, 12(7):654-662.
22. Berry K, Ioannou GN. [Comparison of Liver Transplant-Related Survival Benefit in Patients With Versus Without Hepatocellular Carcinoma in the United States.](http://www.ncbi.nlm.nih.gov/pubmed/26021233) Gastroenterology. 2015 Sep;149(3):669-80.
23. Vitale A, Farinati F, Burra P, et al; Italian Liver Cancer Group. [Utility-based criteria for selecting patients with hepatocellular carcinoma for liver transplantation: A multicenter cohort study using the alpha-fetoprotein model as a survival predictor.](http://www.ncbi.nlm.nih.gov/pubmed/26183802) Liver Transpl. 2015 Oct;21(10):1250-8. doi: 10.1002/lt.24214
24. Lim KC, Wang VW, Siddiqui FJ, et al. Cost-effectiveness analysis of liver resection versus transplantation for early hepatocellular carcinoma within the Milan criteria. Hepatology 2015; 61: 227-37.
25. Spolverato G, Vitale A, Ejaz A, et al. [The relative net health benefit of liver resection, ablation, and transplantation for early hepatocellular carcinoma.](http://www.ncbi.nlm.nih.gov/pubmed/25665675) World J Surg. 2015 Jun;39(6):1474-84. doi: 10.1007/s00268-015-2987-7.
26. Duvoux C, Roudot-Thoraval F, Decaens T, Pessione F, Badran H, Piardi T, et al., Liver Transplantation French Study G. Liver transplantation for hepatocellular carcinoma: a model including alpha-fetoprotein improves the performance of Milan criteria. *Gastroenterology* 2012;**143**(4): 986-994 e983; quiz e914-985.
27. Hameed B, Mehta N, Sapisochin G, Roberts JP, Yao FY. Alpha-fetoprotein level > 1000 ng/mL as an exclusion criterion for liver transplantation in patients with hepatocellular carcinoma meeting the Milan criteria. *Liver transplantation : official publication of the American Association for the Study of Liver Diseases and the International Liver Transplantation Society* 2014;**20**(8): 945-951.
28. Toso C, Meeberg G, Hernandez-Alejandro R, Dufour JF, Marotta P, Majno P, Kneteman NM. [Total tumor volume and alpha-fetoprotein for selection of transplant candidates with hepatocellular carcinoma: A prospective validation.](http://www.ncbi.nlm.nih.gov/pubmed/25777590) Hepatology. 2015 Jul;62(1):158-65. doi: 10.1002/hep.27787. Epub 2015 Apr 22.
29. Yao FY, Mehta N, Flemming J, Dodge J, Hameed B, Fix O, Hirose R, Fidelman N, Kerlan RK Jr, Roberts JP. [Downstaging of hepatocellular cancer before liver transplant: long-term outcome compared to tumors within Milan criteria.](http://www.ncbi.nlm.nih.gov/pubmed/25689978) Hepatology. 2015 Jun;61(6):1968-77. doi: 10.1002/hep.27752. Epub 2015 Mar 20.
30. Hsu CY, Lee YH, Hsia CY, Huang YH, Su CW, Lin HC, Lee RC, Chiou YY, Lee FY, Huo TI. [Performance status in patients with hepatocellular carcinoma: determinants, prognostic impact, and ability to improve the Barcelona Clinic Liver Cancer system.](http://www.ncbi.nlm.nih.gov/pubmed/22806819) Hepatology. 2013 Jan;57(1):112-9. doi: 10.1002/hep.25950.
31. Bolondi L, Burroughs A, Dufour JF, Galle PR, Mazzaferro V, Piscaglia F, Raoul JL, Sangro B. Heterogeneity of patients with intermediate (BCLC B) Hepatocellular Carcinoma: proposal for a subclassification to facilitate treatment decisions. *Seminars in liver disease* 2012;**32**(4): 348-359
